# FLOURISHCARE MODEL OF INTEGRATED CARE: THE VALIDATION OF THE FLOURISH INDEX-REVISED (FI-R)

**DOI:** 10.1093/geroni/igae098.3510

**Published:** 2024-12-31

**Authors:** Anna Faul, Samantha Cotton, Pamela Yankeelov, Barbara Gordon

**Affiliations:** University of Louisville, Louisville, Kentucky, United States; University of Louisville, Louisville, Kentucky, United States; University of Louisvlle, Louisville, Kentucky, United States; University of Louisvlle, Louisville, Kentucky, United States

## Abstract

The FlourishCare (FC) Model for geriatric primary care brings professional disciplines across the continuum of care together to provide quality integrated age-friendly healthcare to older adults with multiple chronic conditions (MCCs). This integrated care model is backed by research demonstrating the efficacy of a comprehensive approach to healthcare by ensuring that services are well-coordinated across the entire spectrum of a person’s health needs. This study validates the Flourish Index–Revised (FI-R), a tool evaluating integrated care models. The FI-R uses 25 quality-of-care indicators and 7 contextual community indicators. The FI-R was validated with Categorial Principal Components Analysis (CATPCA) using a sample of 949 patients 50+ who were mostly female (73%), Non-Hispanic White (70%), living in urban areas (90%), and married (29%), single (22%) or divorced (19%). The mean age was 73.46 (SD=10.86) and mean years of education was 14.30 (SD=2.14). CATPCA showed a four-dimensional structure of biological, psychological, and two social determinants of health (SDOH) subdomains: health behaviors and community. Final selection of indicators was based on total variance accounted for >0.30, absolute values of item loadings >0.45, and not having cross loadings >0.45 on two factors. Internal consistency (Cronbach Alpha) for the determinants were: biological=0.75, psychological=0.76, SDOH: community=0.70, SDOH: Health Behaviors=0.50 and total FI-R=0.95. Sensitivity to change was shown for the total FI-R, psychological determinants, and SDOH:health behaviors but not for biological determinants. The validation of the FI-R shows promise for its usability to evaluate integrated health care models using existing measures in electronic health systems.

